# Immune Escape after Hematopoietic Stem Cell Transplantation (HSCT): From Mechanisms to Novel Therapies

**DOI:** 10.3390/cancers12010069

**Published:** 2019-12-25

**Authors:** Paolo Bernasconi, Oscar Borsani

**Affiliations:** 1 Department of Molecular Medicine, University of Pavia, 27100 Pavia, Italy; 2 Hematology Department, Fondazione IRCCS Policlinico San Matteo, 27100 Pavia, Italy

**Keywords:** acute myeloid leukemia, hematopoietic stem cell transplantation, relapse, donor lymphocyte infusion, hypomethylating agents, mechanisms of immune escape

## Abstract

Acute myeloid leukemia (AML) is the most common type of acute leukemia in adults. Recent advances in understanding its molecular basis have opened the way to new therapeutic strategies, including targeted therapies. However, despite an improvement in prognosis it has been documented in recent years (especially in younger patients) that allogenic hematopoietic stem cell transplantation (allo-HSCT) remains the only curative treatment in AML and the first therapeutic option for high-risk patients. After allo-HSCT, relapse is still a major complication, and is observed in about 50% of patients. Current evidence suggests that relapse is not due to clonal evolution, but instead to the ability of the AML cell population to escape immune control by a variety of mechanisms including the altered expression of HLA-molecules, production of anti-inflammatory cytokines, relevant metabolic changes and expression of immune checkpoint (ICP) inhibitors capable of “switching-off” the immune response against leukemic cells. Here, we review the main mechanisms of immune escape and identify potential strategies to overcome these mechanisms.

## 1. Introduction

In acute myeloid leukemia (AML), the highest priority now is the development of more effective treatments that focus on improving patients’ five-year survival rates, which nowadays are still 30% on average. Poor AML clinical outcomes are predominantly due to disease relapse and are frequently observed in patients treated with chemotherapy alone, despite the possibility of precisely defining the underlying genetic defect and the availability of novel drugs targeting peculiar molecular defects [1]. Thus, for most AML patients allo-HSCT remains the most effective curative option with the best chance of achieving durable complete remission (CR) [2]. However, even with this procedure death due to relapse occurs in about 30–40% of patients depending on the disease type and status at the time of transplant [3,4,5]. Most disease recurrences develop within the first six months after allo-HSCT when patients are still on immune-suppressive treatment. Quick tapering in some clinical conditions may be sufficient to re-induce remission through the so-called graft-versus-leukemia (GvL) effect [6]. This well-known observation is at the basis of the concept that allo-HSCT is a form of immunotherapy in which the intimate cross-talk between host antigen-presenting cells (APC) and donor T cells may truly eradicate the leukemic cell population [7]. Currently, the relevance of this tight interaction has been further substantiated by the demonstration that post-transplant AML relapse is not caused by the development of additional genetic alterations as in patients relapsing after chemotherapy [8], but by the loss or down-regulation of major histocompatibility (MHC) class II genes [9,10]. This seminal finding has paved the way for identifying and revealing other novel potential mechanisms of immune escape that are operative not only in the transplant setting [11,12,13,14], but also in the chemotherapy setting [15,16]. Notably, clarifying studies on these immune escape mechanisms have allowed the development of targeted immunotherapies, which are already providing promising results [11,17]. 

The present review will focus on immune escape mechanisms and potential strategies for their inhibition.

## 2. Mechanisms of Post-Transplant Relapse

### 2.1. Impaired Leukemia Cell Recognition: Loss of Mismatched HLA and Downregulation of HLA Molecules

Haploidentical hematopoietic stem cell transplantation (haplo-HSCT) is considered an effective therapeutic option for patients who need an allo-HSCT, but do not have a fully matched donor. Since its introduction, haplo-HSCT has gained the attention of both hematologists and immunologists because of the complex mechanisms underlying graft-versus-host-disease (GvHD) and the GvL effect. These two post-transplant events are considered “two faces of the same coin” and, to date, there are no well-established methods to switch off the first while retaining the latter. Both of these events rely on T cell alloreactivity, defined as the ability of donor T cells to recognize and target non-self antigens. T cell alloreactivity may be directed against either intact mismatched HLA molecules expressed on recipient cells (i.e., direct T cell alloreactivity) [18] or against peptides derived from proteasomal cleavage of mismatched HLA and presented in the antigen-binding groove of matched HLA molecules (i.e., indirect T cell alloreactivity) [19]. The concept that our immune system discriminates between self and non-self non only by the presence of foreign antigens, but also by the absence of normal self markers has been introduced by Kärre in an elegant work published more than 30 years ago [20]. Recognition of mismatched HLA molecules presented by leukemic stem cells (LSC) is a central function of the GvL immunological mechanism, especially in haplo-HSCT, where considerable genomic differences between the donor and recipient should warrant long-term relapse free survival (RFS) [21]. Human natural killer (NK) cells express multiple receptors that interact with HLA class I molecules. The NK cells’ ability to recognize the absence of self MHC class I is used to discriminate normal cells from cells in distress, like tumor cells. This “missing self” recognition in ensured by inhibitory receptors, which dampen NK cell activation upon interaction with their MHC class I ligands. Consistently, it has been shown that loss of mismatched HLA in the genome of recipient LSC represents an effective mechanism of escape from donor T cell alloreactivity. Genomic loss of mismatched HLA molecules occurs through a mechanism of copy-neutral loss of heterozygosity, which eliminates mismatched HLA alleles without decreasing levels of HLA class I molecule expression. This mechanism of immune escape, also known as immune-selection specific loss, explains why the NK cell-mediated response is not activated. It seems to be especially relevant in the context of allo-HSCT from haploidentical and HLA-mismatched donors where it accounts for more than one-third of relapses [9,22,23,24]. Evidence suggests that this phenomenon may be due to the clonal evolution of LSC as a direct consequence of selective immunological pressure by donor alloreactive T cells [25,26,27]. This assumption is further supported by evidence that the loss of HLA genes is a rare event in HLA-matched allo-HSCT [9,28], where the only targets of donor alloreactive T cells are minor histocompatibility antigens (mHAg) [29]. Therefore, even though there is not clear evidence to support this, it is possible that genomic alterations in mHAg could be the immune escape mechanism operating in HLA-matched HSCT.

The critical role of HLA class-II molecule down-regulation as a mechanism of immune-escape has been further strengthened by other studies that have compared the RNA expression of several genes in paired samples obtained from patients with hematological malignancies at initial presentation and at post-transplant relapse. These studies report the differential expression of more than 200 genes with a known or supposed role in immune function. HLA class-II genes were among the most differentially expressed, with an overall down-regulation at the time of post-transplant relapse [10,30,31]. Consistently, AML cells collected at initial presentation were easily recognized by T cells from HLA-mismatched third-party donors, whereas those collected at post-transplant relapse elicited poor donor T cell activation due to the low expression of HLA class-II molecules. More interestingly, functional in vitro studies revealed that the expression of HLA class-II molecules returned to normal levels when AML cells collected at post-transplant relapse were exposed to interferon-γ (IFN-γ), suggesting that down-regulation of HLA class-II molecules could be dependent on epigenetic mechanisms. This result is supported by the efficacy of hypomethylating agents (HMA), such as 5-azacitidine (AZA) and decitabine, in preventing and treating post-transplant disease relapse [32].

### 2.2. Freezing the Immune Response: Role of Anti-Inflammatory Cytokines

It is well established that leukemic cells can produce anti-inflammatory cytokines to successfully escape immune surveillance [33]. The neoplastic population of chronic myeloid leukemia (CML) cells can produce transforming growth factor (TGF-β) in order to decrease the expression of HLA-class II molecules and in turn become less immunogenic [33,34,35]. This same goal can be achieved by leukemic cells of chronic lymphocytic leukemia (CLL) and AML through the production of interleukin-10 (IL-10) [36,37]. A hypothetical role of interleukin-4 (IL-4) has also been proposed. Some studies have shown that IL-4 production by leukemic cells could promote their immune escape by decreasing the expression of HLA-class II molecules on their surface [33,34,35,36,37]. However, evidence from other studies have demonstrated that IL-4 is able to selectively induce apoptosis of AML cells by upregulating STAT6 target genes [38]. Currently, the exact role of IL-4 in leukemic cell survival is poorly understood and more studies are needed to elucidate the multiple mechanisms of action of this cytokine. 

In addition, leukemic cells and normal hematopoietic progenitors may create an anti-inflammatory microenvironment not only by enhancing the production of anti-inflammatory cytokines, but also by blocking the release of pro-inflammatory cytokines. The low levels of IFN-γ associated with high-risk B-lineage acute lymphoblastic leukemia (B-ALL) have piqued the hypothesis that some leukemic cells might exploit defective IFN-γ production to escape immunological recognition [39]. Even though data derive mainly from studies on B-ALL, it is reasonable that the switching-off of the immune response through the enhanced production of anti-inflammatory cytokines and/or the reduced production of pro-inflammatory cytokines could be a relevant mechanism through which AML cells hamper immune activation after allo-HSCT.

Large amounts of interleukin-15 (IL-15) released from normal myeloid progenitors [40] promote leukemia control after allo-HSCT by boosting T cell alloreactivity against leukemia cells, enhancing NK expansion and instructing the generation of new memory stem T cells from naïve precursors [41,42,43]. Instead, low serum levels of IL-15 and interleukin-7 (IL-7), especially in the early post-transplant period, have been associated with a lower risk of acute GvHD (aGvHD) and a higher risk of relapse; thus supporting the role of these cytokines in improving T cell alloreactivity [44]. Despite the lack of definitive evidence, an anti-tumor role of IL-15 has been suggested by results obtained from a phase I single-arm multicenter study, which reported increased activation and expansion of both CD8+ T cells and NK cells when ALT-803, an IL-15 superagonist complex, was administered to patients who relapsed more than 60 days post-allo-HSCT [43]. Furthermore, studies in animal models have demonstrated that internal tandem duplication (ITD) of the tyrosine kinase FLT3 (FLT3-ITD) negatively regulates transcription of the IL-15 gene through the inhibition of its main transcription regulatory factor: Interferon regulatory factor 7 (IRF7). Thus, AML cells harboring the FLT3-ITD mutation express the IL-15 gene at very low levels. Consistently, the pharmacological inhibition of FLT3 counteracts the IRF7 activation block by reducing the expression of transcription factor ATF4 and restores IL-15 expression. Thus, the inhibition of FLT3, together with T cell transfer, might favor long-term disease control by enhancing the elimination of neoplastic cells [45]. In addition, past studies have demonstrated that IL-15 can also lead to mitochondrial reprogramming, thereby enhancing antineoplastic immunity [46]. As evidenced by human in vitro studies, FLT3 inhibition improves cellular mechanisms responsible for energy production (i.e., glycolysis and mitochondrial respiration), whose functions are essential for CD8+ T cell ATP-dependent cellular activities (e.g., killing of leukemic cells), thereby leading to the enhanced anti-leukemic activity of these immune effectors [45].

To date, it has been well documented that the elimination of apoptotic cells by APC is another important process that is enhanced by anti-inflammatory cytokines (e.g., TGF-β and IL-10). The identification of apoptotic cells by APC involves the recognition of phosphatidylserine (PS) on apoptotic cells by the phosphatidylserine receptor (PSR) expressed on APC. This interaction not only leads to phagocytosis of the apoptotic cell, but also to the production and secretion of anti-inflammatory cytokines. Cancer cells from solid tumors can express both PS and PSR and produce a soluble form of PS (sPS) which interacts and activates the PSR on APC and on neighboring cancer cells, an event that leads to the production and secretion of anti-inflammatory cytokines and facilitates their escape from immune surveillance [47]. Further studies are required to investigate whether this pathway could be exploited by AML cells to hinder their recognition and destruction by immune cells. 

Even though studies are lacking, other humoral factors may also be exploited by leukemia cells to negatively regulate anti-tumor activity. For example, the suppression of Interleukin-1β (IL-1β) and granulocyte-colony stimulating factor (G-CSF) production is a hypothetical mechanism that could be exploited by tumor cells to evade the immune system [12].

### 2.3. Immune-Checkpoint Receptors and Co-Inhibitor Receptors

Under physiologic conditions, T cells receive multiple signals from ICP receptors and these signals are counterbalanced by those provided by co-inhibitory receptors (CTLA-4, PD-1, TIM3, LAG-3, and others). In neoplastic disorders, including AML, co-inhibitory receptors and their ligands may block immune responses by abolishing T cell function and may generate a state of immune tolerance [48]. In AML, this immunological block is due to the fact that despite the presence of a similar number of CD4+ CD8+ T lymphocytes in the leukemic and normal bone marrow (BM), the expression of ICP receptors is higher on T cells contained in the leukemic BM than on T cells contained in the healthy BM [49,50,51].

In addition, various studies have shown that in AML evolved from a myelodysplastic syndrome (MDS) and in relapsed AML, disease aggressiveness directly correlates with increased PD-1 expression on T cells and increased PD-L1 expression on leukemic cells [52,53,54]. A current study has confirmed these findings by revealing a similar percentage of T cells in the BM microenvironment of AML patients and healthy donors, but a higher number of regulatory T (Treg) cells, PD-1-positive/OX40-positive T cells and PD-1-positive/CD8-positive T cells that also express TIM3 or LAG3 in the BM of AML patients [55]. AML patients who experienced multiple relapses or relapsed post-transplant had a higher percentage of PD-1-positive/CD8-positive T cells than that of newly diagnosed or first relapsed patients, and their TP-53-mutated leukemic cells more frequently expressed PD-L1. Gene expression analysis demonstrated that leukemic cells from patients relapsed post-transplant present a deregulated expression of different co-stimulatory molecules and changes in circulating T cells [13]. Interestingly, these patients also have a greater number of early-differentiated memory stem T cells and central memory T cells with multiple inhibitory receptors than those of patients who remain in CR. Moreover, relapse is effectively predicted by the early identification of BM memory stem T cells displaying a severely exhausted phenotype (PD1+, Eomes+, T-bet-) [14].

In this context, therapies aimed at blocking ICP receptors might offer an advantage to prevent and treat AML relapse after allo-HSCT (see Section 3.3 “*Immune Checkpoint Inhibitors*”).

### 2.4. Evasion from NK Cell Recognition and Destruction

Recent studies have revealed that the down-regulation of ligands for NK cell activating receptors or some NK activating receptors and the activation of NK inhibitory receptors may promote AML cell evasion from NK recognition. Leukemic cells may reduce the expression of NK ligands through an aberrant hyper-methylation of genes encoding ligands for the activating receptor NKG2D (NKG2DL), namely MICA, ULBP1, ULBP2, and ULBP3 genes [56], and may also release a soluble form of NKG2DLs (sNKG2DL) which induces the down-regulation of NKG2D receptor [57]. Both of these mechanisms of immune disruption can be removed by HMA, which is thought to block MICA, MICB and ULBP2 shedding by increasing the expression of TIMP3 and may also reduce the release of sNKG2DLs [58]. In addition, AML cells which present ligands for NK activating receptors, namely CD112 and CD155, can decrease the expression of some other NK cell activating receptors, namely DNAM-1, NKp30, and NKp46, by physically interacting with NK cells [59,60]. In particular, leukemic blasts from patients aged less than 65 years may express high levels of CD112 and CD155 in order to decrease DNAM-1 expression on NK cells [61], an event which leads to an altered degranulation of NK cells causing impaired killing of blasts in AML and MDS [62,63]. Thus, it is not surprising that a reduced expression of these activating receptors at AML diagnosis has been associated with a significant and poor prognosis. This has been demonstrated in AML patients with high NKp30 and NKp46 expression, which have a better overall survival (OS) and event-free survival (EFS) than those with low expression [64,65]. In patients submitted to allo-HSCT, a high expression of NKp46 has been associated with a significantly better prognosis [66,67].

AML may also escape NK recognition thanks to the high expression of co-inhibitory receptors including T cell immunoglobulin and immune-receptor tyrosine-based inhibitory motif (ITIM) domain (TIGIT) which inhibit NK cell activity by reducing IFN- γ release, as well as PVRIG and TACTILE, which may act as checkpoint regulators. NK cells, similarly to exhausted CD8+ T cells, may express high levels of TIGIT, which have been correlated with primary refractory AML and leukemia relapse post-HSCT [68]. Currently, in AML patients submitted to allo-HSCT, high TIGIT expression at engraftment has been associated with a reduced number of NK cells in the BM and in the post-transplant follow-up high TIGIT expression is associated with a reduced incidence of aGVHD, poor OS, and progression-free survival (PFS) [69].

Other inhibitory NK receptors include PVRIG, whose only ligand is CD112, and TACTILE, which controls tumor growth/metastasis and DNAM1 activity in mice, but whose role in humans is still debated [70].

### 2.5. Other Mechanisms

Theoretically, the acquisition of new driver mutations that provide a proliferative advantage to leukemia cells could be a mechanism that favors immune escape. Genomic analysis of paired samples collected at initial presentation and at relapse after allo-HSCT have demonstrated that leukemic clones at relapse are genetically different from those present at clinical diagnosis. Most mutations involve genes whose products have a tumor suppressive (e.g., PTEN) or a proliferative (e.g., KRAS) function. Thanks to this proliferative advantage leukemic cells may outnumber the anti-tumor activity of T cells, thereby promoting immune escape by these newly mutated clones [71,72]. 

Leukemia cells can assume specific metabolic features that are useful for impairing T cell functions. For example, kynurenine, a metabolite produced by tryptophan degradation, impairs normal T cell function. T cell function is also impaired in the absence of adequate amounts of tryptophan. Indoleamine 2,3-dioxygenase-1 (IDO1) catalyzes the first, rate limiting step in tryptophan degradation, thereby inversely regulating the amount of tryptophan and of kynurenine. Thus, leukemia cells can negatively regulate T cell function by up-regulating the expression of IDO1 [73]. This is supported by the clinical observation that higher levels of IDO1 expression by leukemia cells correlates with an adverse prognosis in childhood AML [74]. 

CD39 and CD73 are two ectonucleotidases that cooperate in the degradation of adenosine triphosphate (ATP) to adenosine diphosphate (ADP), adenosine monophosphate (AMP), and adenosine. It has been largely demonstrated that adenosine has an immunosuppressive property. Thus, CD39 and CD73 promote an anti-inflammatory microenvironment through the enhanced production of adenosine. Using CD73-defective mice it has been demonstrated that, after allo-HSCT, low CD73 activity enhances leukemia cell recognition and destruction by alloreactive T cells [75,76]. 

Finally, arginine is a basic amino acid whose concentration has a significant impact on T cell function and proliferation. It has been demonstrated that leukemia cells can actively produce and secrete the enzyme arginase, which leads to arginine depletion, thereby impairing proliferation and normal function of T cells and polarizing nearby monocytes and macrophages towards a immunosuppressive M2-like phenotype [77,78,79].

## 3. Potential Treatments

There are several potential treatments aimed at preventing and treating relapse after allo-HSCT. In a schematic and simplified view, they can be classified as drugs or cellular therapies (Table 1).

### 3.1. Tyrosine Kinase Inhibitors (TKI)

TKI are drugs with an intrinsic antitumor effect based on their ability to target tyrosine kinases with aberrant and exaggerated functions selectively expressed by neoplastic clones in a few, specific, hematological malignancies. However, the antitumor effects of TKI also rely on their immunomodulatory effects that allow them to induce T-cell cytolytic functions, reduce PD-1 expression by T-cells and reduce myeloid-derived suppressor cells [80]. 

In patients with CML relapsed after allo-HSCT, anti-BCR-ABL TKI (e.g. imatinib) induce more than 60% of molecular remissions [81], whereas results in Ph+ B-ALL relapsed post-transplant are more controversial [82]. However, their use after allo-HSCT is actually recommended by the European Society for Blood and Marrow Transplantation (EBMT) either as prophylaxis or pre-emptive therapy for MRD-negative Ph+ B-ALL patients [83]. 

In AML the presence of FLT3-ITD at the time of allo-HSCT is predictive of a higher risk of posttransplant relapse (30% vs. 16%) and therapy with anti-FLT3 TKI is of clinical relevance to reduce this risk. Currently, the use of midostaurin, the main FLT3 inhibitor, is approved for AML with mutated FLT3 whereas its use as post-transplant maintenance therapy has been investigated in a phase II clinical trial that reported a 12-month relapse rate of only 9.2% [84]. Sorafenib is another kinase inhibitor that targets a wide range of tyrosine-kinases (e.g., c-KIT, FLT3, VEGFR-2, VEGFR-3, and PDGFR-ß) and serine/threonine-kinases (e.g., BRAF, V600E BRAF, and CRAF) expressed by cancer cells including tumor endothelial cells. Results from animal studies have revealed that the antitumor activity of sorafenib not only relies on its ability to inhibit kinases, but also on its ability to induce IL-15 production thereby enhancing T-cell activation and the GvL effect [43]. A retrospective study that investigated sorafenib as a prophylactic therapy in FLT3-ITD positive AML reported an improved outcome [85]. Currently, other newer anti-FLT3 TKI such as quizartinib, gilteritinib, and crenolanib are under investigation.

### 3.2. Acting on Epigenetic Factors: Hypomethylating Agents and Histone Deacetylase Inhibition

Since methylation is a crucial process involved in the epigenetic control of gene expression, it is not surprising that malignant cells use hypermethylation to switch off the expression of a variety of genes involved in apoptotic cell death and growth inhibition. Given their ability to regulate cell differentiation and cell growth by inhibition of DNA methyltransferase, HMA like AZA and decitabine are currently used to treat MDS and AML. However, several studies have reported that HMA may also upregulate the expression of HLA molecules and tumor associated antigens (TAAs), thus enhancing the ability of donor T cells to recognize, target and kill tumor cells after allo-HSCT [85,86]. In AML/MDS patients with early post-transplant relapse, salvage treatment with AZA or decitabine, administered as single agents or combined with donor lymphocyte infusion (DLI) may lead to 28% CR [87], a result confirmed by a large EBMT study that also reported a low incidence of aGvHD.

Interestingly, various studies have shown that combining AZA with DLI may lead to sustained remissions in about 30% AML/MDS relapsed post-transplant with an incidence and severity of GvHD lower than that occurring after DLI alone [88,89,90]. It has been suggested that this GvHD control may be due to an AZA-dependent upregulation of transcriptional factor FoxP3 and subsequent expansion of CD4+ Treg cells [91,92,93]. Other evidence has shown that administration of AZA to this subset of patients may convert a decreasing donor cell chimerism into a full-donor cell chimerism [94].

However, the most relevant point is that due to their immunomodulation properties and ability to upregulate inhibitory ICP co-receptors on T cells [95], various ongoing clinical trials are currently using HMA in combination with ICP inhibitors to treat relapsed/refractory AML [96]. The rational for this therapeutic strategy was provided by the observation that PD-1, PDL-1, and PDL-2 were more often over-expressed in MDS/AML patients resistant to AZA than in sensitive patients so that the blockade of the PD-1/PDL-1 interaction was supposed to be clinically effective in AZA-resistant patients [53]. One of these ongoing phase Ib/II clinical trials that combined AZA with nivolumab in relapsed/refractory AML achieved a 35% overall response rate (ORR) including 21% CR that were durable in 9/11 patients. In this study CR correlated with a higher pre-treatment BM infiltration of CD3+ and CD8+ T cells and a progressive BM increase of CD4+ and CD8+ T cells during treatment. Instead, in non-responsive patients, a progressive BM increase of CTLA4+ CD8+ T cells during treatment was noted and was thought to flag resistance to treatment [96]. Thus, large prospective randomized clinical trials are currently investigating the role of HMA as treatment able to prevent post-transplant relapse.

Histone deacetylation is another epigenetic mechanism of gene expression control performed by a group of enzymes called Histone Deacetylase (HDAC). HDAC inhibitors upregulate gene expression by unwinding histone-bound DNA. In patients submitted to allo-HSCT this leads to reduced expression of genes involved in inflammatory cytokine production and increases in CD4+ Treg cells, thereby preventing development of GvHD [97]. Panobinostat, an inhibitor of HDAC, was investigated in two different clinical trials as post-transplant maintenance therapy in MDS/AML patients: 1-year RFS was 66% when panobinostat was combined with DLI and 2-year RFS was 74% when panobinostat was administered as a single agent. A prospective, randomized phase III trial testing panobinostat as post-transplant maintenance therapy is currently ongoing.

### 3.3. Immune Checkpoint Inhibitors

Since tumor cells upregulate ICP inhibitors to prevent their killing by immune cells, it has been proposed that Checkpoint Inhibitor inhibition should enhance the GvL effect after allo-HSCT. This pharmacological strategy has already been validated outside the HSCT setting in various cases of solid tumors and hematological malignancies such as Hodgkin lymphoma (HL) with drugs that block CTLA-4 and PD-1 [98]. Moreover, other studies have demonstrated enhanced cytotoxicity in adoptive T-cell therapy by combining PD-1 with cytotoxic T-cell infusions [99]

Two anti-PD-1 antibodies, pidilizumab and nivolumab, have been recently investigated. Pidilizumab, administrated as maintenance therapy after autologous HSCT (auto-HSCT) for diffuse large B cell lymphoma (DLBCL) and nivolumab, administrated as rescue therapy for relapsed HL after auto-HSCT, are well tolerated and ensure a high response rate [100]. However, considering the important T-cell alloreactivity in both the GvHD and GvL effect, there are many concerns regarding the use of anti-PD-1 and anti-CTLA-4 antibodies after allo-HSCT. Another multicenter retrospective study investigated the clinical feasibility and effectiveness of PD-1 blockade with anti-PD-1 antibodies in 31 lymphoma patients with post-transplant relapse: ORR was 77% and aGvHD incidence was 54% with eight patients who died due to GvHD-related complications [101]. In a phase Ib/II clinical trial combining nivolumab with AZA in relapsed/refractory AML patients an ORR of 24% was achieved.

In a phase 1/1b clinical trial, the anti-CTLA-4 antibody ipilimumab was administered at a dose of 3 mg/kg or 10 mg/kg to patients with a variety of hematological cancers (43% of patients had AML) relapsed after allo-HSCT. Among 22 patients who received 10 mg/kg of ipilimumab an ORR of 32% was described, including 22% CR. Grade III-IV aGVHD occurred in 14% patients and was completely resolved with systemic steroid therapy [102].

Extramedullary myeloid leukemia is typically resistant to standard therapies, but provides an antigen-rich environment that can promote T-cell alloreactivity against leukemic cells. Indeed, in one study, all patients with extramedullary leukemia (three with leukemia cutis and one with myeloid sarcoma) achieved complete and durable responses after ipilimumab. The observation that patients who developed GvHD during this treatment were those with a better response to ipilimumab treatment further confirms the intimate relationship between GvHD and GvL. In addition, immunohistochemical and gene-expression analyses revealed that patients who responded to ipilimumab had a higher cytotoxic CD8+ T cell count at the disease site as opposed to those without any response to ipilimumab. The latter presented with an in situ cytotoxic function deficit. This result suggests that ipilimumab resistance may be due to local factors that hamper the acquisition of a cytotoxic phenotype by CD8+ T cells. Moreover, in patients responsive to ipilimumab, peripheral blood analyses revealed the reduction and impaired function of CD4+ Tregs and an increase in CD4+ effector T cells [102]. Further studies combining ipilimumab with decitabine (ClinicalTrials.gov identifier: NCT02890329) in the de novo and post-transplant setting for patients with AML or MDS are currently ongoing.

A phase II nonrandomized study aimed at evaluating the safety and efficacy of AZA with either nivolumab or nivolumab + ipilimumab in relapsed/refractory AML is currently recruiting (ClinicalTrials.gov identifier: NCT02397720). Preliminary results reported a CR rate of 43% in patients receiving AZA plus both nivolumab and ipilimumab (versus a CR rate of 22% in the AZA plus nivolumab arm) [103].

Lastly, a recent phase II single-arm study investigated the safety and efficacy of pembrolizumab, another anti-PD-1 antibody, as induction and maintenance therapy after high-dose cytarabine chemotherapy in patients with relapsed/refractory AML (ClinicalTrials.gov identifier: NCT02768792) and demonstrated a CR rate of 40% [104].

Taken together, these data emphasize the potential role of checkpoint inhibitor blockades to prevent and treat post-transplant relapse, but also underscore the need for further studies aimed at clarifying the potential risks of GvHD. 

### 3.4. Donor Lymphocyte Infusions

Evidence from clinical practice supports a strong link between GvL and GvHD and has revealed that chronic GvHD (cGvHD), but not aGvHD protects patients from disease relapse [105]. Various studies have demonstrated that patients without cGvHD develop disease relapse more often than those with cGvHD of any grade. Thus, many research efforts have tried to separate GvL from GvHD and to identify which immune cell populations are responsible for these post-transplant events. These studies have identified αβ-T cells as the primary immune cells involved in these post-transplant outcomes. αβ-T cells initiate the immune reaction against host tissues and are able to completely eradicate residual, resistant LSC through the GvL effect [106]. 

**Figure 1:**
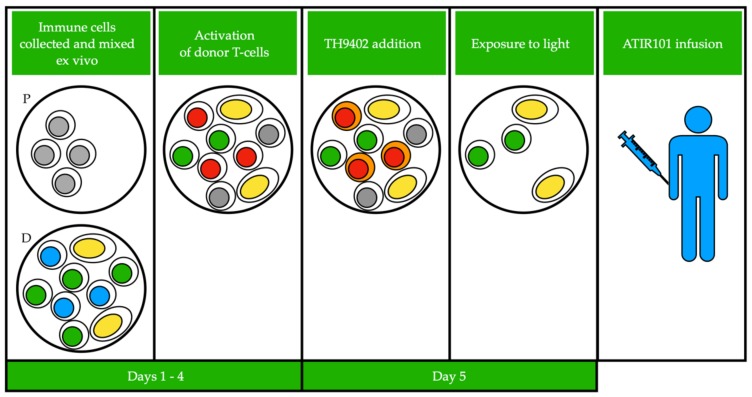
Photodynamic treatment (PDT) of the graft consists of a first phase in which donor and patient peripheral blood mononucleated cells are collected by apheresis. Then, patient cells are gamma-irradiated and donor and patient cells are co-cultured to stimulate activation of host alloreactive T-cells. GvHD causing T-cells from the donor are activated by the presence of “foreign” cells from the patient. TH9402, a photosensitizing reagent, is added to the culture and is retained only in activated donor T-cells. This mixture is then exposed to light, which activates TH9402 leading to production of oxygen radicals, resulting in self-destruction of GvHD causing donor T-cells. The remaining mixture (named ATIR101) is then infused into the patient. Color of the nucleus indicates different cell types: grey, irradiated patient cells; green, virus-specific T cells; blue, inactivated GvHD causing donor T cells; red, activated GvHD causing donor T cells; yellow, other immune cells (e.g., NK cells). Cells that retain the TH9402 reagent have orange colored cytoplasm. P: patient, D: donor.

Based on this seminal knowledge, various procedures including the photodynamic treatment (PDT) of the graft, have been aimed at depleting αβ-T cells from BM or peripheral stem cell grafts in order to prevent GvHD (Figure 1), while other procedures are aimed at exploiting the activity of αβ-T cells in order to prevent post-transplant relapse. 

The demonstration that donor alloreactive T cells are clinically effective in preventing post-transplant relapse was first provided by studies on CML patients in molecular relapse. In 2014, an EBMT study evaluated the sensitivity of different hematological malignancies to the graft-versus-host effects in 48,111 patients submitted to a first allo-HSCT during the period 1998–2007. This study reported that in CML, a reduction in relapse risk was tightly linked with the development and severity of aGvHD and cGvHD and that in both ALL and BCR-ABL negative myeloproliferative disorders (MPD) the relapse risk was similar. In contrast, MDS and lympho-proliferative disorders presented an intermediate correlation with GvHD, while AML and plasma cell disorders had a weak sensitivity to GvHD [107]. These results have been confirmed by a more recent literature review which reported that DLI was successful in 70–80% of patients with hematological/cytogenetic relapse. Patients with multiple myeloma (MM), MDS, and MPD achieved intermediate results, whereas those with acute leukemia, especially ALL, achieved disappointing results [7]. In conclusion, the strength of the GvHD/GvL relationship is different in various hematological disorders. Here we review recent evidence on the efficacy and safety of DLI use in MDS/AML patients relapsing after allo-HSCT. 

Studies have reported that only 15–20% of MDS and AML patients respond to DLI and that these responses have a short duration due to the rapid growth kinetics of the AML cells. Other potential mechanisms responsible for decreased DLI efficacy in AML include lack of surface expression of costimulatory molecules by leukemic cells, involvement of immunologically privileged sites, defective tumor antigen presentation, downregulation of HLA molecules and other patient-specific major and minor histocompatibility antigens [9], suppression by peripheral blood Treg cells [108] and inhibition of NK cell responses [109,110]. Thus, a more recent study has questioned whether DLI may be an adequate treatment option in MDS/AML relapsed after HSCT [111] demonstrating that DLI may be effective when combined with chemotherapy or AZA in patients with low leukemic burden, molecular but not hematological relapse, mixed chimerism and favorable cytogenetics and when the time between allo-HSCT and relapse is longer than five months. In addition, it was observed that an initial CD3+ T-cell dose greater than 1x10^8^/kg was associated with a high GvHD incidence without any improvement of relapse risk and OS. While an interval between allo-HSCT and DLI infusion of less than two years was associated with less GvHD. Thus, it has been proposed that the starting dose of CD3+ T-cells should be between 1 × 10^6^ and 1 × 10^7^/kg and should be escalated until GvHD development [112]. However, despite these results, the timing of DLI has not been established yet. Usually, DLI is performed eight weeks post-transplant and is not administered in the first three months post-transplant except in case of relapse. 

DLI is associated with a 10% mortality rate and may cause pancytopenia in 20% of patients; while GvHD is observed in 60% of patients independently of the grade of HLA donor matching [113]. One study has evaluated pre-emptive DLI (p-DLI) versus DLI performed for late post-transplant relapse (t-DLI) in patients submitted to T-cell depleted HSCT. This study reported an estimated 5-year OS of 80% for p-DLI versus 40% for t-DLI, a 5-year EFS of 65% versus 69% and a 5-year GvHD incidence of 31% versus 41% [114]. Another study treated selected patients (i.e., those who were in CR for at least 120 days from transplantation, off immunosuppression for at least 30 days and free of GvHD) with adjuvant DLI. A 7-year OS of 67% was reported and GvHD was the main clinical complication [115]. 

In conclusion, since all of these studies have provided evidence that DLI produces a GvL effect at the expense of a higher GvHD incidence, various strategies aimed at modifying the DLI content including: Tregs [116,117], NK and γδ-T cells, have been developed [115,116,117].

### 3.5. Antitumor Immunotherapy Based on Dendritic Cells

Dendritic cells (DCs) are professional APC able to uptake, process and present antigens, including TAAs, to naïve CD4+ or CD8+ T-cells in order to activate an immune response against the captured antigen. As such, they represent the principal link between innate and acquired immunity [118]. 

**Figure 2:**
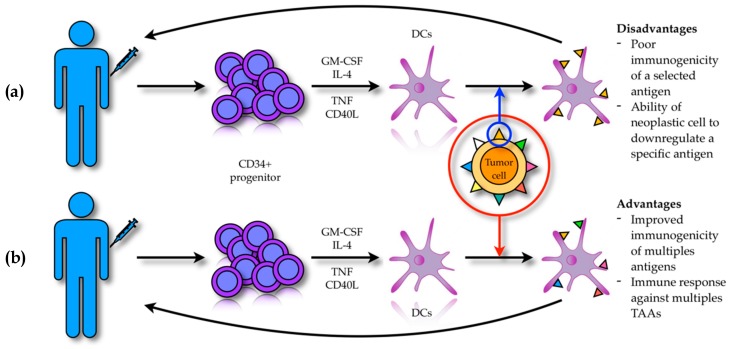
DC-based vaccines exploit the ability of DCs to cross-present TAAs in order to activate a T cell immune response against neoplastic cells. (**a**) CD34+ progenitors obtained from peripheral blood of the patient are exposed in vitro to a mixture of cytokines and differentiate into DCs. These are loaded with a selected target TAA, activated and re-infused back into the patient. However, the poor immunogenicity of the selected TAA and the ability of tumor cells to actively down regulate specific antigen expression under the selective pressure of monoclonal T cells can hinder an effective antitumor response. (**b**) DCs are activated using entire tumor cells: these DCs, when activated and re-infused, are able to stimulate a polyclonal and more effective T cell response against tumor cells.

DCs are normally present within the tumor mass in close association with neoplastic cells, but their anti-tumor response is poor due to the immune-suppressive effect of the tumor milieu, which modulates DC maturation and activity [119,120]. Thus, in order to overcome this problem various research efforts have led to the development of DC-based antitumor vaccines that exploit the ability of DCs to actively infiltrate the tumor mass and cross-present TAAs in order to activate host T-cells and NK-cells [121,122]. DC-based vaccines can be obtained through two distinct strategies (Figure 2). The first strategy (i.e., ex vivo modality), allows DCs to be obtained from patients’ peripheral blood thanks to the expansion of CD34+ progenitor cells cultured in vitro with GM-CSF, IL-4, tumor necrosis factor (TNF), FLT3-ligand and CD40 ligand (CD40L) [123]. These DCs can be loaded with the target TAA, activated and then re-infused back into the patient [124]. However, this strategy is frequently not clinically effective because of the poor immunogenicity of the selected antigen and the ability of the neoplastic cells to down regulate the expression of a specific antigen with consequent immune evasion. In order to overcome these drawbacks, newer approaches based on the in vitro DC activation with entire tumor cells have been developed [125,126]. The rational of this methodology consists in achieving immune activation that is not restricted to a single antigen, but against a broader spectrum of antigens. This strategy might be more effective, allowing an anti-tumor immune response that can also target patient’s specific tumor-associated neo-antigens [127,128].

Deeper knowledge of DC biology has allowed further optimization of DC antigen-presenting functions. Specifically, early clinical trials have reported that activated DCs with a mature phenotype are more capable of activating T-cells. Thus, DCs are now exposed to “maturation cocktails” containing Toll-like-receptor (TLR) ligands, specific pathogen-derived molecules and other “type 1” polarizing agents such as interferons (IFNs). These “maturation cocktails” are aimed at improving DC maturation and activation, thus inducing them to secrete large amounts of IL-12, respond to homing signals from lymph nodes and induce antigen-specific T-cell activation. In a recent study it has been shown that in vitro DC exposure to TLR stimulating agents induces them to differentiate into IFN-producing cells rather than into APCs. In the post-transplant setting, IFN-producing DCs enhance the induction of activated antigen-specific T-cells without stimulating allogeneic T-cells, an event that improves GvL without increasing the risk of GvHD [129]. These results have been confirmed in at least four clinical trials, which demonstrated that immunotherapy with DCs may prevent (or at least delay) AML relapse when CR has been already achieved [130,131,132,133]. DCs vaccines have been evaluated as a form pre-emptive therapy in relapsing AML after allo-HSCT. In a recent phase 1 trial, AML patients with early molecular relapse after allo-HSCT were randomly assigned to receive immunotherapy with either DLI or multi-genetically modified DCs derived from autologous peripheral blood monocytes (moDCs). The vaccine was not only found to be safe, but also induced a three-year OS of 48.9% compared with 27.5% in the DLI group. CR was achieved in 13/23 (57%) patients treated with the moDCs and in 12/25 (48%) treated with DLI. When patients in early molecular relapse (but not in full-blown relapse) were treated with moDCs, a CR rate of 83% was observed, thus indicating that the therapeutic utility of DC vaccines in AML is limited in the case of a high leukemic cell load [133,134].

DC vaccination has been investigated in several other hematological (e.g., MM, non-Hodgkin’s lymphoma) and non-hematological (e.g., malignant melanoma, prostate cancer, renal cell carcinoma, lung, colon, and breast carcinomas) tumor types; however, these studies go beyond the scope of this paper.

### 3.6. Antitumor Strategies Based on NK Cells

NK cells have been recognized as a critical cellular component of the innate immune system [135,136,137,138,139,140,141]. This is based on their ability, after receiving activating stimuli, to kill target cells and to secrete cytokines involved in shaping and guiding both the innate and the adaptive immune response to infections and malignant transformation.

Among NK receptors, one of the most studied are the killer immunoglobulin-like receptors (KIR), which consist of at least fifteen different receptors that map to chromosome 19q13.4 [142,143,144]. KIRs recognize peculiar domains of class I HLA alleles. Depending on the intracellular tyrosine-based motif of the molecules, KIRs may be either activating or inhibitory [142]. Simultaneous binding of both activating and inhibiting KIRs generally inhibits NK cells from killing [145].

While T cell activity against tumor cells depends on the recognition of specific TAAs, NK cell rejection of tumor cells occurs independently of TAA recognition. This property has been exploited by different NK cell-based immunotherapies. Among these, the first and simplest procedure to be applied was the direct infusion of NK cells into patients. This strategy was based on the observation that patients who received allogeneic peripheral blood stem cell grafts containing a higher amount of NK cells had better clinical outcomes in terms of post-transplant infections and non-relapse mortality [146,147]. In one clinical trial, direct NK cell infusions in the haploidentical allogeneic setting was associated with longer RFS [148]. However, in the absence of data from randomized clinical studies demonstrating a survival advantage after NK infusion in AML patients relapsed after allo-HSCT, there are no current indications concerning the use of this therapy outside of clinical trials.

Another NK cell-based immunotherapy consisted of the in vivo expansion of NK cells obtained from leukemic patients after the administration of various recombinant human cytokines including IL-2 at low, intermediate, and high doses [149,150,151]. This strategy was further improved upon by the administration of a tumor specific antibody whose Fc portion was able to bind CD16 expressed on NK cells thereby enhancing the so called antibody-dependent cellular cytotoxicity (ADCC) [151,152,153]. 

A third NK cell-based immunotherapy was developed with more in depth knowledge of KIR biology [154]. Several years ago, it was observed that the killing activity of NK cells was inversely correlated with the expression of HLA class I molecules on target cells [20]. This breakthrough led to the so called “missing self” hypothesis, which nowadays represents the main mechanism by which NK cells are thought to operate. Subsequently, three main HLA class I allele specificities were identified: “group 1” comprising HLA-C alleles which express Asn80, “group 2” comprising HLA-C alleles which express Lys80 and “group 3” comprising HLA-Bw4 alleles (e.g., HLA-B27). The NK receptor repertoire is dictated and shaped during NK cell development and maturation by the HLA class I genotype in a such way that every mature NK cell expresses at least one inhibitory KIR specific for self HLA class I molecules [142]. Thus, allogenic targets sensitive to NK cytotoxicity are identified by their lack of self HLA class I inhibitory KIR ligands. Despite some conflicting data, it might be possible that, in the allo-HSCT setting, this donor-versus-recipient NK alloreactivity could improve engraftment and protect against GvHD with enhanced recipient survival and in haplo-HSCT it may protect against relapse [110,155].

Retention of HLA class I molecules by tumor cells represents a mechanism of immune escape and has become the target of many strategies. Among these, the manipulation of the relationship between NK receptor and HLA class I molecules with monoclonal antibodies seems to be the most promising [156,157]. 

### 3.7. Chimeric Antigen Receptor T Cell Therapies

The remarkable success of chimeric antigen receptor (CAR)-T cells in lymphoproliferative disorders has fueled enthusiasm over developing a similar therapeutic strategy in AML. However, the identification of unique and disease-specific target antigens for CAR-T cells in AML is much more difficult, as myeloid leukemic cells, including LSC, present antigens that are also expressed on normal hematopoietic progenitors. Thus, it is not surprising that CAR-T are still considered investigational treatments in AML due to their toxicity in normal hematopoiesis.

Despite this drawback, some antigens have already been tested as potential targets in various on-going clinical trials. Among them, CD33, a member of the sialic acid-binding Ig-like lectin family, is the most attractive as it is expressed in 90% of adult and pediatric AMLs [158]. Anti-CD33 monoclonal antibodies have proved their clinical efficacy in a peculiar subset of AML patients [159] and preclinical studies with anti-CD33 CAR-T have achieved promising results despite a severe concomitant myelotoxicity [160]. To date, the only patient with relapsed/refractory AML who received anti-CD33 CAR-T developed cytokine release syndrome (CRS), experienced severe pancytopenia and relapsed nine weeks after the CAR-T cell infusion [161]. Currently, various research groups supported by the observation that in animal models CD33 is not absolutely required for normal hematopoietic function [162], have developed different genetic approaches to inactivate CD33 on hematopoietic stem cells (HSC) in order to avoid the severe myelotoxicity associated with anti-CD33 CAR-T infusions and have reported promising results [163,164,165]. 

CD123, the alpha chain of the interleukin-3 receptor, is another suitable target but, like CD33, its targeting is associated with severe myelotoxicity [166]. However, a very recent study has revealed that in high-risk MDS, CD123 can effectively distinguish between normal HSCs and MDS stem cells and anti-CD123 CAR-T can selectively kill MDS stem cells in vitro [167]. 

Since FLT3 mutations occur in about 20% of AML patients, FLT3 has been identified as another potential CAR-T target despite the fact that it is also expressed on HSCs and multipotent hematopoietic progenitors [168]. Interestingly, it has been reported that in AML patients carrying the FLT3-ITD mutation, the therapeutic efficacy of anti-FLT3 CAR-T can be dramatically increased with crenolanib, due its ability to increase FLT3 expression on the leukemic cell surface [169]. 

However, the most promising CAR-T target might be CD44v6 as it is uniquely and specifically expressed on leukemic cells [170,171]. A preclinical study carried out in immunocompromised mice has demonstrated that second generation anti-CD44v6 CAR-T cleared AML and multiple myeloma cells, spared normal HSC and keratinocytes, but determined severe monocytopenia [172]. Regarding this last construct, the dose-limiting effect as well as hyper-acute xenogeneic GvHD were circumvented by the introduction of thymidine kinase [173] and inducible Caspase 9 suicide genes [174] into these CAR-T cells to effectively eliminate them within a few hours. 

Since the C-type lectin-like molecule 1 is another antigen exclusively expressed on the surface of AML cells, it may also be a potential CAR-T target [175,176].

## 4. Conclusions

Currently, the demonstration that about two-thirds of post-transplant relapses are caused by unique patterns of immune escape that might be targeted by specific therapies has diminished the conventional wisdom, which for several years has assumed that alloreactive donor T-cells may cure AML patients. 

In the near future, this seminal knowledge will require that in post-transplant relapse all potential mechanisms of immune escape should be accurately investigated in order to decide on the best therapeutic procedure for every single patient. Thus, a new type of personalized medicine is now emerging thanks to studies on post-transplant relapse, but its clinical effectiveness will depend on the assessment of precise biological criteria.

## Figures and Tables

**Table 1:** Schematic view of the main drugs and cellular therapies used to prevent and treat relapse after allo-HSCT.

Therapeutic Strategy		Mechanisms of Action	Examples
**Drugs**	TKI	Intrinsic antitumor activity by inhibition of abnormal tyrosine kinases and other kinases Boost T-cell cytolytic functions Reduction of PD-1 expression by T-cells Reduction of myeloid-derived suppressor cells Induction of IL-15 production	imatinib midostaurin quizartinib gilteritinib crenolanib sorafenib
	HMA	Regulation of cell differentiation and cell growth Up-regulation of HLA and TAA on neoplastic cells, thus improving cellular immune responses against them Reduction of GvHD risk by up-regulation of FoxP3 and subsequent expansion of regulatory T cells	azacytidine decitabine
	HDACi	Down-regulation of genes involved in production of inflammatory cytokines Expansion of regulatory T cells	panobinostat
	ICP inhibitors	Promotion of T cell responses against tumor cells	nivolumab ipilimumab pidilizumab
**Cellular Therapies**	DLI	Direct antitumor activity derived from infused donor T cells	
	DC infusion	Stimulation of antitumor cellular response by enhancing DC ability to process and present TAA to host T cells	Sipuleucel-T
	NK cell based therapies	Stimulation of antitumor cellular responses by direct infusion of either un-manipulated NK cells or IL-2 pre-treated NK cells Promotion of tumoral lysis by antibody-dependent cellular cytotoxicity by administration of antibodies with a double specificity for TAA expressed on neoplastic cells and CD16 expressed on NK cells Use of anti-KIR antibody to disrupt KIR-HLA interaction and improve NK activation Use of bivalent proteins with a double specificity for both NKG2D activating receptor on NK cells and CD138 on myeloma cells	ULBP2-BB4
	CAR-T cell based therapies	Intrinsic antitumoral activity based on ability to recognize specific TAAs and activate T cell cytolytic program against tumor cells	

CAR: chimeric antigen receptor, DC: dendritic cells, DLI: donor lymphocyte infusion, HMA: hypomethylating agents, HDACi: inhibitors of histone deacetylase, ICP: immune-checkpoint, NK: natural killer, TAA: tumor associated antigens, TKI: tyrosine kinase inhibitors.

## Abbreviations

ADCC	antibody-dependent cellular cytotoxicity
ADP	adenosine diphosphate
aGvHD	acute graft-versus-host disease
AMP	adenosine monophosphate
ALL	acute lymphoblastic leukemia
Allo-HSCT	allogeneic hematopoietic stem cell transplantation
AML	acute myeloid leukemia
APC	antigen presenting cells
ATP	adenosine triphosphate
auto-HSCT	autologous hematopoietic stem cell transplantation
AZA	5-azacytidine
BM	bone marrow
CAR	chimeric antigen receptor
CD40L	CD40 Ligand
cGvHD	chronic graft-versus-host disease
CLL	chronic lymphocytic leukemia
CML	chronic myeloid leukemia
CR	complete remission
CRS	cytokine release syndrome
DC	dendritic cells
DLBCL	diffuse large B cell lymphoma
DLI	donor lymphocyte infusion
EBMT	European Society for Blood and Marrow Transplantation
EFS	event-free survival
G-CSF	granulocyte-colony stimulating factor
GvHD	graft-versus-host disease
GvL	graft-versus-leukemia
Haplo-HSCT	haploidentical hematopoietic stem cell transplantation
HDAC	histone deacetylase
HL	Hodgkin lymphoma
HMA	hypomethylating agents
HSC	hematopoietic stem cell
ICP	immune-checkpoint
IDO1	indoleamine 2,3-dioxygenase-1
IFN	interferon
IL	interleukin
IRF7	interferon regulatory Factor 7
ITD	Internal Tandem Duplications
ITIM	Immune-receptor Tyrosine-based Inhibitory motif
KIR	killer immunoglobulin-like receptors
LSC	leukemic stem cells
MDS	myelodysplastic syndrome
mHAg	minor histocompatibility antigens
MHC	major histocompatibility
MM	multiple myeloma
moDCs	multi-genetically modified DCs derived from autologous peripheral blood monocytes
MPD	myeloproliferative disorder
NK	natural killer
NKG2DL	NKG2D ligand
ORR	overall response rate
OS	overall survival
p-DLI	preemptive donor lymphocyte infusion
PFS	progression-free survival
PS	phosphatidylserine
PSR	phosphatidylserine receptor
RFS	relapse-free survival
sNKG2DL	soluble form of NKG2DL
sPS	soluble form of Phosphatidylserine
TAA	tumor associated antigens
t-DLI	tardive donor lymphocyte infusion
TGF	transforming growth factor
TIGIT	T cell immunoglobulin and ITIM domain
TKI	tyrosine kinase inhibitors
TLR	Toll-like receptor
TNF	tumor necrosis factor
Treg	regulatory T cell.
